# Low Th2 and high PD1^+^ TFh cells in blood predict remission after CTLA-4Ig treatment for 48 weeks in early rheumatoid arthritis

**DOI:** 10.1371/journal.pone.0330823

**Published:** 2025-08-28

**Authors:** Tilia Selldén, Kerstin Andersson, Inger Gjertsson, Anna-Karin Hultgård Ekwall, Kristina Lend, Merete Lund Hetland, Mikkel Østergaard, Tillmann Uhlig, Marte Schrumpf Heiberg, Michael T. Nurmohamed, Jon Lampa, Tuulikki Sokka Isler, Dan Nordström, Kim Hørslev-Petersen, Bjorn Gudbjornsson, Gerdur Gröndal, Ronald van Vollenhoven, Cristina Maglio, Anna-Carin Lundell, Anna Rudin

**Affiliations:** 1 Department of Rheumatology and Inflammation Research, The Sahlgrenska Academy at University of Gothenburg, Gothenburg, Sweden; 2 Department of Rheumatology, Sahlgrenska University Hospital, Gothenburg, Sweden; 3 Department of Rheumatology and Clinical Immunology, Amsterdam University Medical Center, Amsterdam Rheumatology and Immunology Center, Amsterdam, The Netherlands; 4 Department of Medicine, Rheumatology Unit, Center for Molecular Medicine, Karolinska Institute, Stockholm, Sweden; 5 Copenhagen Center for Arthritis Research, Center for Rheumatology and Spine Diseases, Centre for Head and Orthopaedics, Rigshospitalet, Copenhagen, Denmark; 6 Department of Clinical Medicine, University of Copenhagen, Copenhagen, Denmark; 7 Center for Treatment of Rheumatic and Musculoskeletal Diseases (REMEDY), Diakonhjemmet Hospital, Oslo, Norway; 8 Amsterdam Rheumatology and immunology Center, Amsterdam, The Netherlands; 9 Jyväskylä Central Hospital, University of Eastern Finland, Jyväskylä, Finland; 10 Division of Internal Medicine and Rheumatology, Helsinki University Hospital and University of Helsinki, Helsinki, Finland; 11 Department of Rheumatology, Danish Hospital for Rheumatic Diseases, Sønderborg, Denmark; 12 Department of Regional Health Research, University of Southern Denmark, Odense, Denmark; 13 Landspitali University Hospital, University of Iceland, Reykjavik, Iceland; 14 Department of Rheumatology, Centre for Rheumatology Research, Reykjavik, Iceland; University of South Florida, UNITED STATES OF AMERICA

## Abstract

**Objective:**

To determine whether baseline CD4^+^ T helper (Th) cell subset proportions in blood may serve as predictive biomarkers for achieving remission 48 weeks after initiating CTLA-4Ig, anti-tumor necrosis factor (TNF), or anti-interleukin 6 receptor (IL6R) treatment in patients with early rheumatoid arthritis (eRA).

**Methods:**

This study included 60 untreated eRA patients from the larger randomized treatment trial NORD-STAR. They were treated with methotrexate (MTX) combined with either CTLA-4Ig (n = 17), anti-TNF (n = 22), or anti-IL6R (n = 21). Disease activity was assessed by clinical disease activity index (CDAI), C-reactive protein, and erythrocyte sedimentation rate. The primary outcome was remission (CDAI ≤ 2.8) at week 48, and the secondary outcomes were time to reach remission or sustained remission during the 48-week follow-up. CD4^+^ T cell subset proportions were analyzed fresh by flow cytometry at baseline and at 24 and 48 weeks.

**Results:**

In CTLA-4Ig + MTX-treated patients, baseline Th2 together with PD1^+^ T follicular helper (TFh) cell proportions predicted CDAI remission at week 48 (AUC: 0.986, 95% CI 0.94–1.0). Survival analysis revealed that patients with Th2 proportions below 16.8% or PD1^+^ TFh proportions above 7.6% at baseline were more likely to achieve remission (log-rank *p = *0.002 and *p = *0.007, respectively), and sustained remission (log-rank *p = *0.01 and p = 0.001, respectively), over the 48-week follow-up. CD4^+^ T cell subset proportions did not predict remission in patients treated with anti-TNF + MTX or anti-IL6R + MTX. Only CTLA-4Ig treatment reduced PD1^+^ TFh and PD1^neg^ TFh fractions after 48 weeks.

**Conclusion:**

Circulating Th2 and PD1^+^ TFh cell proportions at baseline may serve as predictive biomarkers for achieving CDAI remission after 48 weeks of CTLA-4Ig treatment in eRA.

## Introduction

Rheumatoid arthritis (RA) is a systemic autoimmune disease characterized by synovial inflammation and progressive joint and bone destruction if left untreated. In RA, CD4^+^ T helper (Th) cells are important players in initiation and progression [1]. Genes associated with T cell activation are enriched in the synovial tissue of seropositive individuals at risk of arthritis development, as well as in individuals with established RA [2,3]. A CD4^+^ T cell-driven disease is further supported by the clinical effectiveness of cytotoxic T-lymphocyte-associated protein 4 (CTLA-4)Ig that blocks Th cell co-stimulation in untreated early RA (eRA) patients [4,5], and its inhibitory effect on RA development in individuals at high risk [6,7]. However, the distribution of CD4^+^ T cell subpopulations can vary considerably among patients with eRA, influenced by factors such as age and sex [8,9].

The heterogeneity in immune phenotype is likely linked to treatment response in eRA patients. Although biological disease-modifying anti-rheumatic drugs (bDMARDs) have markedly improved clinical outcomes in individuals with RA, a substantial proportion of eRA patients fail to achieve remission within the initial year of bDMARD treatment [4,5]. As some patients respond to certain treatments but not to others, there is a need for early quantitative biomarkers to guide the optimal choice of therapy. We have previously shown that specific circulating CD4^+^ T cell proportions could predict clinical remission at 24 weeks in eRA patients treated with methotrexate (MTX) combined with CTLA-4Ig, but not with MTX combined with anti-tumor necrosis factor (TNF) or anti-interleukin 6 receptor (IL6R) [10]. We found that high proportions of T follicular helper (TFh) cells expressing programmed cell death 1 (PD1) and CD4^+^ T cells expressing CTLA-4 predicted remission after CTLA-4Ig treatment. In established previously treated RA patients, circulating proportions of CD4^+^ T cells, including TFh, T regulatory (Treg), and CXCR3^+^ Th17 cells, have been reported to predict therapeutic response to CTLA-4Ig treatment after 24 weeks [11–13]. However, it is unknown whether CD4^+^ T cell subset proportions may predict remission also after 48 weeks of bDMARD treatment in patients with untreated eRA.

Treatment with bDMARD differentially alters the composition of CD4^+^ T cells in the blood of RA patients. In patients with established RA, treatment with CTLA-4Ig for 52 weeks reduces the proportions of activated TFh, Th1Th17, and Treg cells [12,14]. In patients with DMARD-naïve eRA, we have shown that MTX combined with CTLA-4Ig, anti-TNF, or IL-6R decreased the proportions of Treg and Th17 cells, while solely CTLA-4Ig reduced the PD1^+^ TFh proportions after 24 weeks compared to baseline [10]. However, the effects of treatment with different bDMARDs on circulating CD4^+^ T cell subset proportions in blood after 48 weeks have never been studied.

In the present study, we included 60 previously DMARD-naïve eRA patients treated head-to-head with MTX in combination with either CTLA-4Ig, anti-TNF, or anti-IL6R as part of a randomized treatment trial [4,5]. We aimed to evaluate whether CD4^+^ T cell subsets at baseline could predict remission after 48 weeks of treatment, as well as sustained remission. As previous reports, including our own, have shown that TFh cells that express PD1 predict therapeutic response to CTLA-4Ig, we included additional CD4^+^ Th cell subtypes that express PD1 in this study. Moreover, we also analyzed the effects of the different bDMARDs on blood CD4^+^ T cell subset proportions after 48 weeks of treatment.

## Patients and methods

### Study population

This prospective study included 60 DMARD-naïve eRA patients recruited at rheumatology clinics at Sahlgrenska University Hospital (n = 46) or Skåne University Hospital (n = 14), Sweden, as a part of the NOrdic Rheumatic Diseases Strategy Trials And Registries (NORD-STAR) study, a phase four investigator-initiated, randomized, observer-blinded clinical trial, with four treatment arms followed during 48 weeks of treatment (NORD-STAR Trial registration number: EudraCT 2011-004720-35; ClinicalTrials.gov NCT01491815). We chose to exclude the prednisolone + MTX arm in this study since it did not include a specific immunological target. Recruitment took place in the period March 19^th^, 2013 – September 18^th^, 2018. All patients met the American College of Rheumatology (ACR)/The European Alliance of Associations for Rheumatology (EULAR) 2010 criteria for RA diagnosis and were DMARD and glucocorticoid naïve at inclusion. Inclusion criteria were: ≥ 18 years of age, ≥ 2 swollen joints (66 joint count), ≥ 2 tender joints (68 joint count), disease activity score 28 with C-reactive protein (DAS28-CRP) of ≥3.2, and a retrospective patient-reported ymptom duration of <24 months. Patients were required to be rheumatoid factor (RF)-positive or anti-citrullinated antibody (ACPA)-positive or have a CRP level ≥ 10 mg/L. The study was conducted in compliance with the Helsinki Declaration and approved by the regional ethics committees of Stockholm (Dnr. 2011/2069–31/4) and Gothenburg (Dnr. 691–12 and amendment T270-13). All study participants provided written informed consent to participate.

### Study format and clinical evaluation

The complete NORD-STAR study protocol and primary clinical outcome results at 4, 12, 24, and 48 weeks have been published elsewhere [4,5,15]. All patients received MTX escalated to 25 mg/week within the first 4 weeks, combined with either CTLA-4Ig (abatacept; Bristol Myers Squibb, New York City, NY, USA), anti-TNF (certolizumab-pegol; UCB, Brussels, Belgium), or anti-IL6R (tocilizumab; Hoffmann-La Roche, Basel, Switzerland). Oral steroids were not allowed. Disease activity was assessed before treatment initiation (baseline) and at 4, 12, 24, and 48 weeks after initiation by swollen joint counts in 28 and 66 joints (SJC28 and SJC66), tender joint counts in 28 and 68 joints (TJC28 and TJC68), patient global score of disease activity (PGA) and physician global score of disease activity (PhGA), CRP, erythrocyte sedimentation rate (ESR), DAS28, and clinical disease activity index (CDAI). CDAI is calculated as the sum of SJC28, TJC28, PGA, and PhGA measurements. ACPA and RF positivity were determined at baseline by a multiplexed anti-CCP test (BioPlex from BioRad, Hercules, CA, USA) and nephelometry (Beckman Coulter, Brea, CA, USA), respectively, and positivity was determined according to cutoff levels in the local clinical immunology laboratories. The 48-week primary clinical outcome was the achievement of remission (defined as CDAI ≤ 2.8). Blood samples analyzed in this study were drawn at baseline (within 1–2 weeks after RA diagnosis) and after 24 and 48 weeks of treatment.

### Flow cytometry

**Fig 1:**
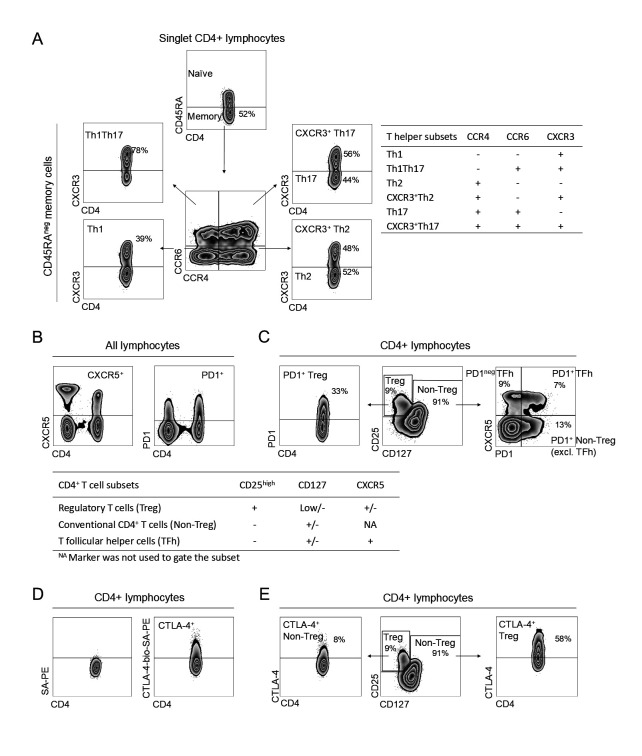
Gating strategy for CD4^+^ T cell subsets. **(A)** Memory Th cell subsets, i.e., Th1, Th1Th17, CXCR3^+^Th17, Th17, CXCR3^+^Th2 and Th2, were defined based on the expression of CCR4, CCR6, and CXCR3. Th cell subset fractions are presented as proportions of memory CD45RA^neg^ cells. **(B)** CXCR5 and PD1 positive gates were based on their expression on CD4^neg^ and CD4^+^ T cells among lymphocytes. **(C)** Treg cells were defined as CD25^high^CD127^low/neg^, and the remaining conventional CD4^+^ T cells were defined as Non-Treg. PD1^+^ cells were gated among Tregs and Non-Tregs (excluding TFh cells), respectively. TFh was defined as CXCR5^+^ Non-Tregs and further divided into PD-1^neg^ TFh or PD1^+^ TFh. **(D)** Positivity for CTLA-4 was determined by comparing the control that contained the SA-PE fluorophore but not the CTLA-4-biotin antibody to the stained sample. **(E)** CTLA-4-positive cells were gated among Tregs and Non-Tregs, respectively. All Treg and Non-Treg cell subset fractions are presented as proportions of CD4^+^ cells. Parts of this gating strategy have previously been described [10]. TFh, T follicular helper; Th, T helper; Treg, T regulatory; SA, streptavidin.

Blood CD4^+^ T cell subset proportions were analyzed using flow cytometry at the Clinical Immunology Laboratory and Transfusion Medicine at the Sahlgrenska University Hospital in Gothenburg. The results for weeks 0, 4, 12, and 24 have been published by our group previously [10]. All samples were analyzed fresh since freeze-thaw cycles affect the expression of several markers used to categorize T cell subsets [10]. In brief, peripheral blood mononuclear cells (PBMCs) were isolated using Lymphoprep (Axis-Shield, Oslo, Norway) and stained with fluorochrome-conjugated antibodies (listed in S1 Table). Absolute CD4^+^ T cell counts were assessed using TruCount™ assay (BD Biosciences, Becton, Dickinson U.K). For the detection of intracellular expression, cells were fixed and permeabilized. All samples were acquired on a FACS Canto II (FACS Diva; BD Biosciences), with daily quality control using BD FACSDiva™ CS&T IVD beads (BD Biosciences) to ensure consistent performance of the flow cytometer. No significant batch effects were found using this protocol [10]. Flow cytometry data were analyzed blindly by K.A. using FlowJo (Tree Star, Ashland, OR, USA). The following T cell subsets were analyzed as proportions and as numbers: Th1, Th1Th17, Th2, CXCR3^+^ Th2, Th17, CXCR3^+^ Th17, PD1^+^ TFh and, PD1^neg^ TFh and PD1 and/or CTLA-4 positive Treg, and PD1 and/or CTLA-4 positive conventional CD4^+^ T cells (Non-Treg excluding TFh). We have previously demonstrated that the above-mentioned CD4^+^ Th cell subsets can be defined by distinct chemokine receptor expression based on expression of transcription factors and cytokine secretion profiles [8]. Treg were defined as CD25^high^CD127^low/neg^, which we and others have demonstrated to be suppressive in vitro [16,17]. The gating strategy is previously described in detail [10], and also presented in Fig 1.

### Statistical analysis

Multivariate factor analysis (SIMCA-P + , version 18.0.0; Umetrics, Umeå, Sweden) by orthogonal projection to latent structures discriminant analysis (OPLS-DA) was used to investigate the relationship between baseline CD4^+^ T cell subset proportions and achieving remission (CDAI ≤ 2.8, yes/no) at week 48. Each variable was log-transformed, centered, and scaled by unit variance scaling. The quality of each model was measured by R2 (i.e., how well the variance of the data is explained by the model) and Q2 (i.e., how well the model predicts the variable data). Subsequently, univariate statistical analyses using the Mann-Whitney U test (GraphPad Prism, version 10.2.0, La Jolla, CA, USA) were applied only to variables that contributed most to each model to avoid multiple testing.

Logistic regression, presented as the area under the curve (AUC) in receiver operating characteristic curves (ROC curves), was used to test whether specified baseline CD4^+^ T cell subset proportions predict remission at week 48 (Graphpad Prism). Positive predictive value (PPV) and negative predictive value (NPV) were calculated based on the remission rate with probability cutoff values that maximize specificity and sensitivity. The optimal sensitivity and specificity were calculated based on the Youden Index (R with RStudio, PBC, Boston, MA; R package pROC).

Time to reach remission as well as sustained remission, defined as achieved remission at two or more consecutive visits up to 48 weeks, was assessed by Kaplan-Meier estimates of cumulative incidence rates and compared between binary groups. Patients were stratified based on (1) the optimal probability cutoffs and (2) the median baseline CD4^+^ T cell subset proportions (low: proportions ≤ median; high: proportions > the median). Kaplan–Meier curves were compared with log-rank tests. Hazard ratio (HR) and 95% confidence intervals (CIs) were calculated using Cox regression models (R packages: survival, survminer). The Exact method was used for estimation of ties, and visualization of the Schoenfeld residuals against time was used to assess whether the proportional hazards assumption was fulfilled in each Cox regression model.

Principal component analysis (PCA) and OPLS (SIMCA-P+) were used to assess the association between the baseline CD4^+^ T cell subsets proportions and the clinical parameters at week 48. Correlation analyses, Spearman’s rank correlation test (GraphPad Prism), were applied only to variables that contributed most to the OPLS model. Correlation strength was determined by the correlation coefficient (r) as weak (0.2 ≤ r < 0.4), moderate (0.4 ≤ r < 0.6), strong (0.6 ≤ r < 0.8), or very strong (r ≥ 0.8). Pairwise comparisons were performed using the Wilcoxon matched pairs signed rank test or the Friedman test, followed by Dunn’s multiple comparison test (GraphPad Prism) as described in the respective figure legends. Patients with missing data at one or more time points were excluded from the pairwise comparisons. Statistical significance was defined as p ≤ 0.05 (* *p* ≤ 0.05, ** *p* ≤ 0.01, *** *p* ≤ 0.001 and **** *p* ≤ 0.0001). The data analyzed in the present study have been published as an independent data publication in a data repository with restricted access [18], doi: https://doi.org/10.5878/t95r-4t86.

## Results

**Fig 2:**
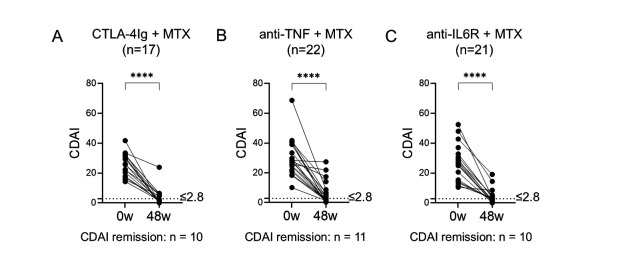
Treatment reduces the clinical disease activity index after 48 weeks. RA disease activity measured by the CDAI at baseline (0w) and 48w in patients treated with MTX in combination with **(A)** CTLA-4Ig (abatacept, n = 17), **(B)** anti-TNF (certolizumab-pegol, n = 22), or **(C)** anti-IL6 receptor (tocilizumab, n = 21). The dotted line indicates the cutoff value for remission (CDAI ≤ 2.8). Paired Wilcoxon matched pairs signed rank test, **** *p* ≤ 0.0001. Two patients with missing data in the anti-TNF + MTX group and four patients with missing data in the anti-IL6R + MTX group at 48w are shown in the graph but not included in the pairwise comparisons. 0w, 0 weeks/baseline; 48w, 48 weeks; anti‐IL6, anti–interleukin‐6 receptor; anti‐TNF, anti–tumor necrosis factor; CDAI, clinical disease activity index; MTX, methotrexate.

Baseline demographics and clinical characteristics of the untreated eRA patients who received MTX in combination with either CTLA-4Ig, anti-TNF, or anti-IL6R have been previously published [10] and are shown in S2 Table. Baseline CRP, ESR, and the percentage of double seronegative (ACPA^neg^ RF^neg^) patients were significantly higher in the anti-TNF arm compared to the CTLA-4Ig and anti-IL6R arms [10]. None of the other assessed baseline variables differed significantly between the treatment arms. In all treatment arms, CDAI (Fig 2A-C) as well as SJC66, TJC68, PGA, PhGA, ESR, and CRP (S1A-F Fig) decreased significantly from baseline to week 48.

### Circulating proportions of Th2 in combination with PD1^+^ TFh at baseline predict remission in eRA patients treated with CTLA-4Ig

**Fig 3:**
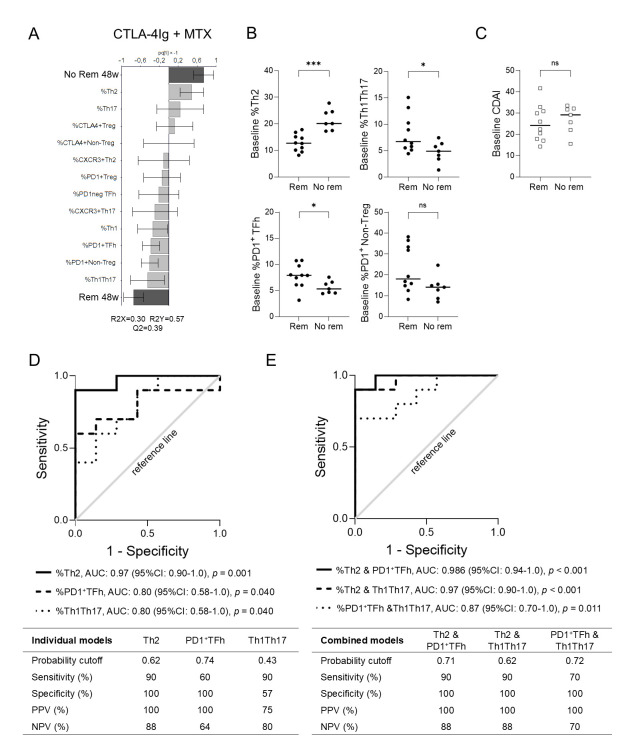
Baseline CD4^+^ T cell subset proportions predict remission in patients treated with CTLA-4Ig. **(A)** Orthogonal projection to latent structures discriminant analysis of Rem (CDAI ≤ 2.8) at 48w (binary Y-variable) and baseline Th cell subset proportions (X-variables) in CTLA-4Ig + MTX-treated patients (n = 17). **(B)** Comparison of baseline Th2, Th1Th17, PD1^+^ TFh, and PD1^+^ Non-Treg proportions in patients who did or did not achieve Rem at 48w. **(C)** Comparison of baseline CDAI in patients who did or did not achieve Rem at 48w. Mann-Whitney U-test, *p ≤ 0.05 and ***p ≤ 0.001. Bars indicate median. ROC curves of baseline Th2, PD1^+^ TFh, and Th1Th17 proportions in **(D)** individual regression models, or **(E)** in combined multiple regression models. Probability cutoffs were determined by maximizing the Youden index for each model. 0w, 0 weeks/baseline; 48w, 48 weeks; anti‐IL6, anti–interleukin‐6 receptor; anti‐TNF, anti–tumor necrosis factor; AUC, area under the curve; CDAI, clinical disease activity index; MTX, methotrexate; NPV, negative predictive value; PPV, positive predictive value; PD1, programmed cell death‐1; Rem, remission; ROC, receiving operating curve; TFh, T follicular helper; Th, T helper.

We first investigated whether baseline circulating CD4^+^ T cell subset proportions could predict remission (CDAI ≤ 2.8, yes/no) at week 48 in each treatment arm. In the CTLA-4Ig + MTX treatment arm, OPLS-DA showed that failure to achieve remission at week 48 was associated with higher Th2 proportions, while achieving remission was associated with higher Th1Th17, PD1^+^ Non-Treg and PD1^+^ TFh proportions (Fig 3A). Univariate analysis showed that Th2 proportions were significantly higher in patients who did not achieve remission after 48 weeks compared to patients who did, while proportions of Th1Th17 and PD1^+^ TFh cells, but not PD1^+^ Non-Treg, were significantly higher in the remission group (Fig 3B). There was no difference in baseline CDAI scores between the two groups (median 29.2 and 24.2, *p = *0.6; Fig 3C). In ROC analysis, the AUC for the proportions of Th2 cells, PD1^+^ TFh, and Th1Th17 at baseline were 0.97, 0.80 and 0.80 (95% CI 0.90–1.0, 0.58–1.0 and 0.58–1.0), respectively (Fig 3D). When Th2 and PD1^+^ TFh subsets were combined, the AUC increased to 0.986 (95% CI 0.94–1.0) compared to the individual models, with a sensitivity of 90% and a specificity of 100% (Fig 3E). In the anti-TNF and anti-IL6R treatment arms, the OPLS-DA models showed that CD4^+^ T cell subset proportions at baseline were not predictive for achieving remission at 48 weeks (S2A-D Fig).

**Fig 4:**
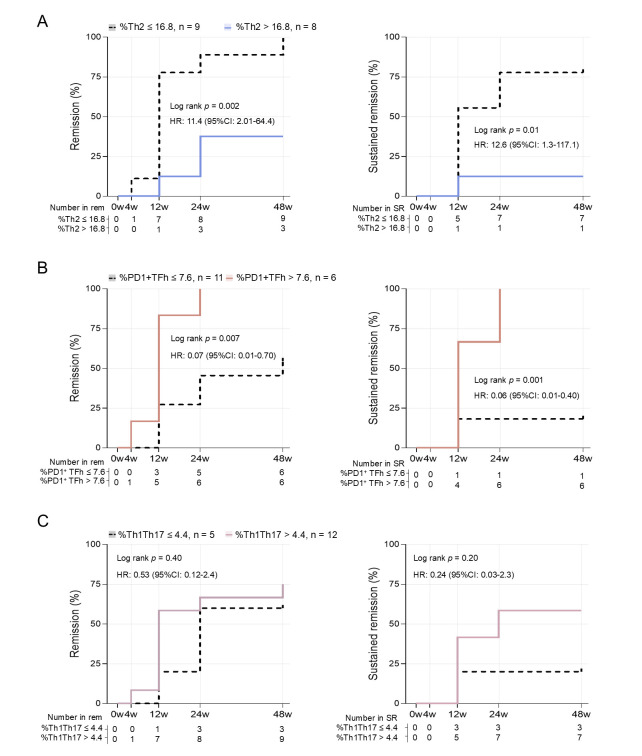
Baseline CD4^+^ T cell subset proportions are associated with time to achieve remission in CTLA-4Ig-treated patients. Kaplan-Meier cumulative incidence analysis showing the percentage of patients in remission (CDAI ≤ 2.8) and in sustained remission, i.e., CDAI remission for two or more consecutive visits, with **(A)** Th2 proportions ≤ 16.8% (dotted line) or > 16.8% (blue line), and **(B)** PD1^+^ TFh proportions ≤ 7.6% (dotted line) or > 7.6% (orange line), and **(C)** Th1Th17 proportions ≤ 5.8% (dotted line) or > 4.4% (pink line) at baseline. The groups for the Kaplan-Meier estimates were determined by the probability threshold that maximizes the Youden index. Significance was tested with the log-rank test, and unadjusted HR for achieving remission were estimated using Cox regression. Reference groups were patients with low Th2, PD1^+^ TFh, or Th1Th17 proportions. Each Cox regression model fulfilled the proportional hazards assumption. CDAI, clinical disease activity index; CI, confidence interval; HR, hazard ratio; PD1, programmed cell death‐1; Rem, remission; SR, sustained remission; TFh, T follicular helper; Th, T helper.

We subsequently examined the time to achieve remission in the CTLA-4Ig + MTX treatment arm over 48 weeks. Patients were stratified based on the optimal probability cutoff values identified in the ROC analyses (Fig 3D), which were 16.8% for Th2, 7.6% for PD1^+^ TFh, and 4.4% for Th1Th17, respectively. As shown in Fig 4A, patients with Th2 proportions ≤ 16.8% were more likely to achieve remission (log-rank *p = *0.002 and corresponding unadjusted HR: 11.4, 95% CI 2.01–64.4), as well as sustained remission (log-rank *p = *0.01 and corresponding unadjusted HR: 12.6, 95% CI 1.3–117.1) compared to those with Th2 proportions > 16.8%. Patients with PD1^+^ TFh proportions > 7.6% were more likely to achieve remission (log-rank *p = *0.007 and corresponding unadjusted HR: 0.07, 95% CI 0.01–0.70), and sustained remission (log-rank *p = *0.001 and corresponding unadjusted HR: 0.06, 95% CI 0.01–0.40) compared to those with proportions < 7.6% (Fig 4B). The Th1Th17 proportions at baseline were unrelated to time to achieve remission (Fig 4C). Using a median split as cutoff values, groups showed similar differences in time to achieve remission (log-rank *p* = 0.002, *p* = 0.05, and *p* = 0.9 for Th2, PD1^+^ TFh, and Th1Th17, respectively; S3A-C Fig). There were no significant differences in age, ESR, or CDAI at baseline between patients with low Th2 or PD1^+^ TFh proportions compared to high proportions, but symptom duration and CRP differed between patient groups (S3 Table). To summarize, our findings suggest that baseline Th2 in combination with PD1^+^ TFh proportions may predict remission at week 48 in eRA patients treated with CTLA-4Ig + MTX, but not in those treated with MTX in combination with anti-TNF or anti-IL6R. Furthermore, patients with Th2 proportions ≤ 16.8% or PD1^+^ TFh proportions > 7.6% at baseline were more likely to achieve remission and sustained remission over the 48-week follow-up period.

### Lower disease activity at week 48 after CTLA-4Ig treatment is associated with lower Th2 and higher PD1-expressing Th subset proportions at baseline

**Fig 5:**
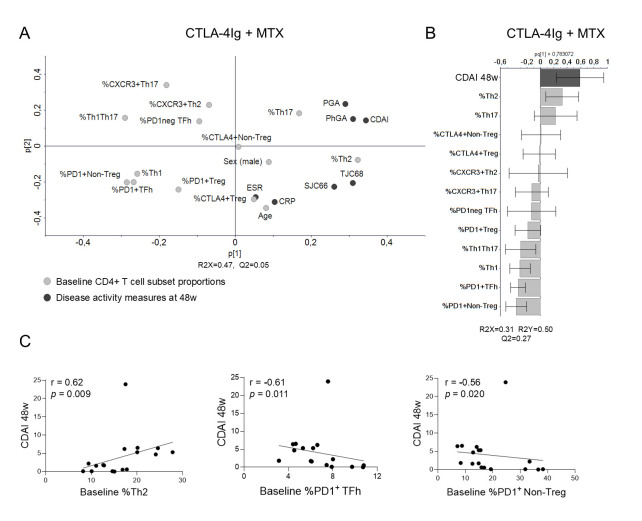
Association of baseline CD4^+^ T cell subset proportions and disease activity after 48 weeks of CTLA-4Ig treatment. **(A)** Unsupervised PCA of disease activity measures at 48w, patient demographics, and baseline T cell subset proportions (X-variables) in CTLA-4Ig + MTX-treated patients (n = 17). **(B)** Orthogonal projection to latent structures analysis of CDAI at 48w (Y-variable) and baseline Th cell subset proportions (X-variables). **(C)** Unadjusted Spearman’s rank correlation test and simple linear regression analyses of CDAI at 48w and the Th2, PD1^+^ Non-Treg, and PD1^+^ TFh proportions at baseline. 48w, 48 weeks; MTX, methotrexate; PD1, programmed cell death‐1; PCA, principal component analysis; TFh, T follicular helper; Th, T helper; Treg, T regulatory.

Next, we investigated the association between baseline CD4^+^ T cell subset proportions and the different disease activity measurements at week 48 in CTLA-4Ig-treated patients. PCA showed that CDAI, PGA, PhGA, TJC68, and SJC66 clustered together with Th2 and Th17 proportions, but opposite to most other Th proportions (Fig 5A). OPLS analysis confirmed positive associations between CDAI and Th2 proportions and negative associations with PD1^+^ Non-Treg, PD1^+^ TFh, Th1, and Th1Th17 proportions (Fig 5B). In univariate analyses, we found a strong positive correlation between CDAI and Th2 proportions (r = 0.62, *p = *0.009), as well as strong or moderate inverse correlations respectively between CDAI and PD1^+^ TFh and PD1^+^ Non-Treg proportions (r = −0.61, *p = *0.011 and r = −0.56, *p = *0.020; Fig 5C). Exclusion of one patient with very high CDAI at week 48 improved the correlation coefficients in all univariate analyses (r = 0.63, *p = *0.010, r = −0.70, *p = *0.003, and r = −0.73, *p = *0.002 for Th2, PD1^+^ TFh, and PD1^+^ Non-Treg proportions, respectively). Separate OPLS analyses of PGA, PhGA, TJC68, and SJC66 at week 48 all showed positive associations with Th2 proportions (S4 Fig). Thus, lower disease activity measurements after 48 weeks of CTLA-4Ig + MTX treatment are associated with low Th2 and high PD1^+^ TFh and PD1^+^ Non-Treg cell proportions at baseline.

### Treatment with CTLA-4Ig reduces circulating proportions of PD1-expressing CD4^+^ T cell subsets

**Fig 6:**
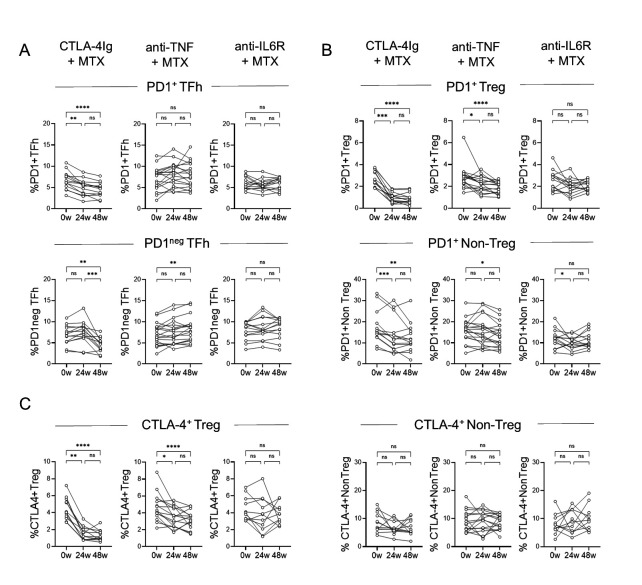
Treatment for 48 weeks distinctly affects CD4^+^ T cell subset proportions. Comparison of the proportions of circulating **(A)** PD1^+^ TFh and PD1^neg^ TFh, **(B)** PD1-expressing Non-Treg (excluding TFh) and Treg, and **(C)** CTLA-4-expressing Non-Treg and Treg at 0w and at weeks 24 and 48 in patients treated with MTX + CTLA-4Ig (abatacept, n = 17), MTX + anti-TNF (certolizumab-pegol, n = 22), or MTX + anti-IL6 receptor (tocilizumab, n = 21), respectively. Friedman’s test with Dunn’s test for multiple comparisons. *p ≤ 0.05, **p ≤ 0.01, ***p ≤ 0.001 and ****p ≤ 0.0001. Patients with missing data for one or more time points were not included in the pairwise comparisons. 0w, 0 weeks/baseline; 24w, 24 weeks; 48w, 48 weeks; anti‐IL6, anti–interleukin‐6 receptor; anti‐TNF, anti–tumor necrosis factor; MTX, methotrexate; PD1, programmed cell death‐1; TFh, T follicular helper; Th, T helper; Treg, T regulatory.

Finally, we investigated the effect of treatment with MTX in combination with CTLA-4Ig, anti-TNF, or anti-IL6R on CD4^+^ T cell subset proportions and numbers in eRA patients before and after 24 and 48 weeks of treatment. CTLA-4Ig treatment significantly reduced PD1^+^ TFh proportions between baseline and week 48, whereas anti-TNF and anti-IL6R treatment did not affect the proportions of this cell type (Fig 6A). Regarding PD1^neg^ TFh, CTLA-4Ig treatment resulted in decreased proportions and anti-TNF treatment in increased proportions at 48 weeks (Fig 5A). Treatment with CTLA-4Ig or with anti-TNF resulted in reduced fractions of PD1^+^ Treg, PD1^+^ Non-Treg (excluding TFh), and CTLA-4^+^ Treg from baseline to 48 weeks, while anti-IL6R had no effect (Fig 6B-C). None of the treatments affected CTLA-4^+^ Non-Treg proportions after 48 weeks compared to baseline (Fig 6C). Analyses of CD4^+^ T cell subset cell numbers resulted in similar results as observed for proportions (S5 Fig).

**Fig 7:**
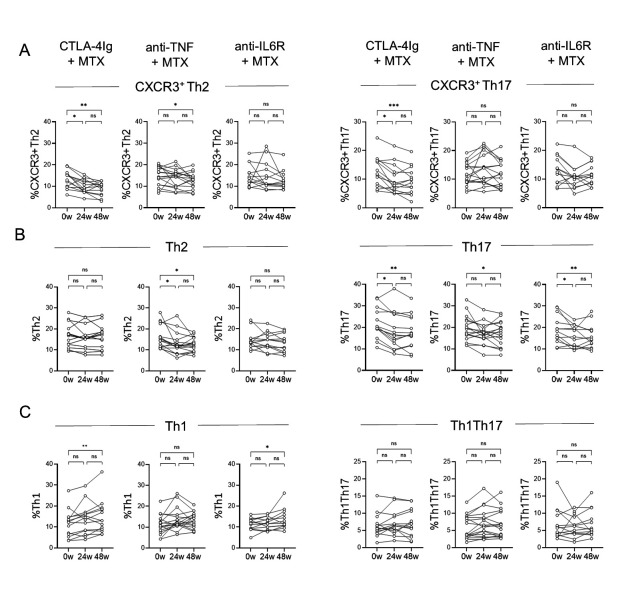
Treatment for 48 weeks distinctly affects T-helper cell subset proportions. Comparison of the proportions of **(A)** CXCR3^+^ Th2 and CXCR3^+^ Th17 and **(B)** Th2 and Th17 and **(C)** Th1 and Th1Th17 of CD45RAneg memory cells in blood at 0w and 24w and 48w in patients treated with MTX + CTLA-4Ig (abatacept, n = 17), MTX + anti-TNF (certolizumab-pegol, n = 22), or MTX + anti-IL6 receptor (tocilizumab, n = 21), respectively. Friedman’s test with Dunn’s test for multiple comparisons. * *p* ≤ 0.05, ** *p* ≤ 0.01 and ***p ≤ 0.001. Patients with missing data for one or more time points were not included in the pairwise comparisons. Data for 0w and 24w have previously been published [10]. 0w, 0 weeks/baseline; 24w, 24 weeks; 48w, 48 weeks; anti‐IL6, anti–interleukin‐6 receptor; anti‐TNF, anti–tumor necrosis factor; MTX, methotrexate; PD1, programmed cell death‐1; TFh, T follicular helper; Th, T helper; Treg, T regulatory.

We have previously shown that treatment with bDMARDs differentially alters the proportions of CXCR3^+^ Th2, CXCR3^+^ Th17, and Th1Th17 as well as classical Th cells, including Th1, Th2, and Th17 after 24 weeks [10]. After 48 weeks of treatment with CTLA-4Ig, the proportions of CXCR3^+^ Th2 and CXCR3^+^ Th17 cells had decreased (Fig 7A). Treatment with anti-TNF decreased the proportions of CXCR3^+^ Th2 and Th2 cells, while all treatments reduced the proportions of Th17 cells (Fig 7A- B). Moreover, both CTLA-4Ig and anti-IL6R increased the proportions of Th1, but Th1Th17 proportions were unaffected by the different bDMARD treatments (Fig 7C). As for CD4^+^ T cell subset cell numbers, CTLA-4Ig treatment reduced the CXCR3^+^ Th2, CXCR3^+^ Th17 cell counts and increased the Th1 cell numbers in blood between baseline and 48 weeks (S6A-C Fig). Finally, anti-TNF treatment, but not CTLA-4Ig and anti-IL6R, increased total CD4^+^ T cell number in blood (S7 Fig).

## Discussion

A considerable proportion of eRA patients fail to achieve remission, despite treatment with bDMARD as a first line of therapy, as there is no biomarker available to predict treatment effectiveness in an individual to a specific treatment [4,5,19–21]. In this spin-off study of a randomized treatment trial, we demonstrated that Th2 and PD1^+^ TFh proportions at baseline were predictive of CDAI remission at 48 weeks in patients treated with the Th cell co-stimulation blocker CTLA-4Ig, but not in those treated with anti-TNF or anti-IL6R. Moreover, the study design allowed for longitudinal analysis in which we found that low Th2 proportions or high PD1^+^ TFh proportions at baseline favor earlier achievement of CDAI remission after CTLA-4Ig treatment. Only CTLA-4Ig treatment reduced the fraction of PD1^+^ TFh and PD1^neg^ TFh cells at week 48 compared to baseline, whereas both CTLA-4Ig and anti-TNF reduced circulating PD1-expressing conventional CD4^+^ T cell and Treg proportions.

We have previously shown that PD1^+^ TFh and CTLA-4^+^ Non-Treg proportions in blood may serve as biomarkers for the effect of CTLA-4Ig treatment at 24 weeks in untreated eRA [10]. Here, we confirm that PD1^+^ TFh cell proportions at baseline predicted remission also at 48 weeks in patients treated with CTLA-4Ig, but not in those treated with anti-TNF or anti-IL6R. Our findings are consistent with observations in established RA, where high TFh proportions at baseline have been shown to predict clinical response to CTLA-4Ig treatment at 24 weeks [11], as well as after 52 weeks of therapy [14]. In addition, CTLA-4Ig is more effective in ACPA/RF-positive compared to seronegative RA patients [22–24], which may be explained by the fact that TFh cells, that are specialized in supporting B cell activity [25,26] are expanded in the lymph nodes from seropositive eRA patients compared to healthy controls [27]. Thus, circulating PD1^+^ TFh proportions may mirror the overactivity in germinal centers seen in eRA, which can be restrained by CTLA-4Ig treatment. Notably, the proportions of other PD1-expressing Th subtypes, including PD1^+^ Non-Treg and PD1^+^ Treg, were not associated with CDAI remission after CTLA-4Ig + MTX treatment. In this study, however, PD1^+^ Non-Treg were not further stratified into specific Th subtypes, and thus it cannot be ruled out that specific Th subtypes, other than TFh, that express PD1 may be predictive for CTLA-4Ig + MTX treatment.

In addition to PD1^+^ TFh, low baseline Th2 proportions emerged as a new predictive marker for remission at week 48 in treatment naïve eRA patients treated with CTLA-4Ig in the present study. One previous report in established RA found no association of Th2 cell proportions at baseline and response to CTLA-4Ig [13]. This discrepancy may be explained by prior treatment history, as more than 30% of the patients in that study had bDMARD treatment at inclusion [13]. In eRA patients treated with MTX monotherapy, Th2-like cells in blood at baseline were less frequent in responders compared to non-responders [28]. The role of Th2 cells in RA is still only partly understood. Th2 cells are expanded in the blood of DMARD-naïve eRA patients compared to age- and sex-matched healthy controls and may be a feature of systemic RA [8]. Also, Th2 cytokines, including IL-4 and IL-13, are elevated in the synovial fluid of individuals with early inflammatory arthritis who subsequently develop RA [29]. However, Th2 cells have been shown to have anti-inflammatory functions in animal models of arthritis [30,31]. This has not been confirmed in RA, as treatment blocking IL-4 and IL-13 seems to protect against RA in a pharmacovigilance study [32]. In the present study, the patients with low Th2 proportions did not have lower disease activity at baseline compared with the patients with high Th2 proportions, which could otherwise have explained the higher remission rates in these patients. Circulating Th2 proportions could be an indirect marker of proportions of T cell subtypes in the joint that we did not measure in the present study. In contrast to PD1^+^ TFh cell proportions, we show that circulating Th2 cell proportions are unaffected by CTLA-4Ig + MTX treatment at 48 weeks compared to baseline.

To the best of our knowledge, this is the first study to demonstrate that specific CD4^+^ T cell subtypes are associated not only with treatment outcomes but also with the time to achieve remission. Notably, over 75% of patients with either below-threshold Th2 proportions or above-threshold PD1^+^ TFh proportions at baseline achieved CDAI remission within 12 weeks of treatment with CTLA-4Ig + MTX. In contrast, baseline proportions of Th1Th17 cells were unrelated to time to achieve remission. The large variability in baseline Th1Th17 proportions within the groups likely contributes to this lack of a clear relationship. In established RA, it has been reported that baseline proportions of Th1Th17 were, on the other hand, inversely correlated with the therapeutic response to CTLA-4Ig [13]. Prior bDMARD treatment history in that study may contribute to discrepancies between the two studies.

Baseline proportions of CD4^+^ T cell subtypes were not associated with remission after 48 weeks of treatment with MTX combined with either anti-TNF or anti-IL6R therapy. This may be because TNF or IL-6R blockade does not modulate CD4^+^ T cell subtype distributions as extensively as CTLA-4Ig treatment. To identify predictors of response to anti-TNF and anti-IL6R therapies, profiling of other immune cell populations, such as monocytes, may be needed. Among the three bDMARDs assessed herein, anti-TNF therapy has been shown to exert the most pronounced effect on monocyte activation [33].

Only treatment with CTLA-4Ig + MTX reduced PD1^+^ TFh and PD1^neg^ TFh proportions between baseline and 48 weeks of treatment. Likewise, reduced TFh proportions have been reported after treatment with CTLA-4Ig for 24 weeks in eRA [10,34], and after treatment with CTLA-4Ig for 52 weeks in established RA [14]. A novel finding in the present study was that CTLA-4Ig and anti-TNF treatment regimens also reduced PD1^+^ CD4^+^ T cell (excluding TFh) and PD1^+^ Treg proportions after 48 weeks. The reduced inflammatory activity may contribute to the decrease in these PD1^+^ CD4^+^ subset proportions. Moreover, as others have shown that MTX monotherapy did not affect the PD1^+^ CD4^+^ T cell proportions after six months of treatment in DMARD-naive eRA patients [28], the specific mode of action of CTLA-4Ig and anti-TNF may contribute to the reduction in PD1^+^ CD4^+^ T cell proportions. Our observations indicate that the reduction in circulating PD1^+^ CD4^+^ T cell proportions was most pronounced after CTLA-4Ig treatment compared to anti-TNF, which could be attributed to the fact that CD28 engagement is crucial for PD1 surface expression on conventional CD4^+^ T cells [35].

Strengths of this study are that all patients included were untreated and had newly diagnosed RA, which eliminates differences in disease duration and treatment history, and that we compare the effects of three different bDMARDs prospectively on circulating CD4^+^ T cell subset proportions in a randomized clinical trial. Furthermore, by analyzing freshly isolated PBMC, we avoid the potential impact of freezing and/or ex vivo stimulation, factors that might have contributed to variability in T cell data in previous studies. An important limitation of the study is that it includes a relatively small number of patients in each treatment arm which may limit the statistical power and generalizability of the findings. As such, our findings should be interpreted with caution and need to be validated in a larger, independent cohort of patients with eRA. Moreover, while our findings offer insight into how bDMARD treatment affects CD4^+^ T cell subtypes in the blood, they do not account for the potential treatment effects on joint-resident T cells which play a critical role in the initiation and progression of joint inflammation in RA.

## Conclusion

The data herein show that low Th2 and high PD1^+^ TFh cell proportions in blood could serve as potential biomarkers for achieving remission with CTLA-4Ig treatment in eRA patients. If these findings are replicated in a larger cohort, it may facilitate the identification of patients likely to benefit from early CTLA-4Ig treatment and enable timely intervention with this effective but expensive therapy.

## Supporting information

S1 Table Antibodies used for flow cytometry. (TIF)

S2 Table Baseline characteristics of untreated early RA patients in each respective treatment arm. (TIF)

S3 Table Baseline characteristics of untreated early RA patients in the CTLA4-Ig + MTX treatment arm. (TIF)

S1 Fig Treatment for 48 weeks reduces disease activity. RA disease activity measured by **(A)** the swollen joint count 66 (SJC66), **(B)** tender joint count 68 (TJC68), **(C)** patient global assessment (PGA), **(D)** physician global assessment (PhGA), **(E)** ESR, and **(F)** CRP at baseline (0w) and at 48 weeks (48w) in patients treated with methotrexate (MTX) + CTLA-4Ig (abatacept, n = 17), MTX + anti-TNF (certolizumab-pegol, n = 22), or MTX + anti-IL6 receptor (tocilizumab, n = 21), respectively. Wilcoxon matched-pairs signed rank test ***p ≤ 0.001 and ****p ≤ 0.0001. Patients with missing data for w48 are not included in the pairwise comparisons.
(TIF)

S2 Fig Baseline CD4^+^ T cell subset proportions do not predict remission in patients treated with anti-TNF or anti-IL6R. OPLS-DA column loading plots showing the association between remission at week 48 (binary Y-variable) and T-cell subset proportions in blood at baseline (X-variables) in patients treated with **(A)** methotrexate (MTX) + anti-TNF or **(B)** MTX + anti-IL6R. (C-D) Comparison of circulating proportions of Th2 and Th1 at baseline in patients who did or did not achieve remission (CDAI ≤ 2.8) at week 48 in the MTX + anti-TNF or MTX + anti-IL6R treatment arm, respectively. Mann–Whitney U-test. Bars indicate median. Two patients with missing data in the MTX + anti-TNF group and six patients with missing data in the MTX + anti-IL6R group were excluded from the analyses.
(TIF)

S3 Fig Baseline CD4 + T cell subset proportions are associated with time to achieve remission in CTLA-4Ig-treated patients. Kaplan-Meier cumulative incidence analysis showing the percentage of patients in remission and in sustained remission, i.e., CDAI remission for two or more consecutive visits, with **(A)** Th2 proportions below the median (≤ 16.8%, dotted line) or above (> 16.8%, blue line) or **(B)** PD1^+^ TFh proportions below median (≤ 6.6%, dotted line) or above (> 6.6%, orange line), and **(C)** Th1Th17 proportions below median (≤ 5.8%, dotted line) or above (> 5.8%, pink line) or at baseline. Significance was tested with the log-rank test, and unadjusted hazard ratios (HR) for achieving remission were estimated using Cox regression. Reference groups were patients with Th2, PD1^+^ TFh, or Th1Th17 proportions below the median. 95%CI: 95 percent confidence interval. Each Cox regression model fulfilled the proportional hazards assumption.
(TIF)

S4 Fig Association of baseline CD4^+^ T cell subset proportions and disease activity after 48 weeks of CTLA-4Ig treatment. OPLS-DA column loading plots showing the association between patient global assessment (PGA), physician global assessment (PhGA), tender joint count-68 (TJC68) or swollen joint count-66 (SJC66) at 48w (Y-variables) and T-cell subset proportions in blood at baseline (X-variables) in patients treated with methotrexate (MTX) + CTLA-4Ig.
(TIF)

S5 Fig Treatment for 48 weeks distinctly affects CD4^+^ T cell subset counts. Comparison of the cell count per µL blood of **(A)** PD1^+^ TFh and PD1^neg^ TFh, **(B)** PD1-expressing conventional CD4^+^ T cells (Non-Tregs, excluding TFh) and regulatory T cells (Tregs), and **(C)** CTLA-4-expressing Tregs and Non-Tregs at baseline (0w), at 24 weeks, and 48 weeks in patients treated with methotrexate (MTX) + CTLA-4Ig (abatacept, n = 17), MTX + anti-TNF (certolizumab-pegol, n = 22), or MTX + anti-IL6 receptor (tocilizumab, n = 21), respectively. Friedman’s test with Dunn’s test for multiple comparisons. *p ≤ 0.05, **p ≤ 0.01, ***p ≤ 0.001 and ****p ≤ 0.0001.
(TIF)

S6 Fig Treatment for 48 weeks distinctly affects T-helper cell subset counts. Comparison of the cell count per µL blood of **(A)** CXCR3^+^ Th2 and CXCR3^+^ Th17, **(B)** Th2 and Th17, and **(C)** Th1 and Th1Th17 at baseline (0w), at 24 weeks and 48 weeks in patients treated with methotrexate (MTX) + CTLA-4Ig (abatacept, n = 17), MTX + anti-TNF (certolizumab-pegol, n = 22), or MTX + anti-IL6 receptor (tocilizumab, n = 21), respectively. Friedman’s test with Dunn’s test for multiple comparisons. *p ≤ 0.05, **p ≤ 0.01 and ***p ≤ 0.001.
(TIF)

S7 Fig Absolute counts of CD4 + T cells after treatment for 48 weeks. Comparison of the absolute counts of CD4^+^ T cells at baseline and at 24 and 48 weeks in the blood of patients treated with **(A)** methotrexate (MTX) + CTLA-4Ig (abatacept, n = 17), **(B)** MTX + anti-TNF (certolizumab-pegol, n = 22), or **(C)** MTX + anti-IL6 receptor (tocilizumab, n = 21). Friedman’s test with Dunn’s test for multiple comparisons. *p ≤ 0.05 and **p ≤ 0.01.
(TIF)
