# Supplementary figures and images for: Low Th2 and high PD1+ TFh cells in blood predict remission after CTLA-4Ig treatment for 48 weeks in early rheumatoid arthritis
Source: PLoS One. 2025 Aug 28;20(8):e0330823. doi: 10.1371/journal.pone.0330823 (PMC12393762; doi:10.1371/journal.pone.0330823)

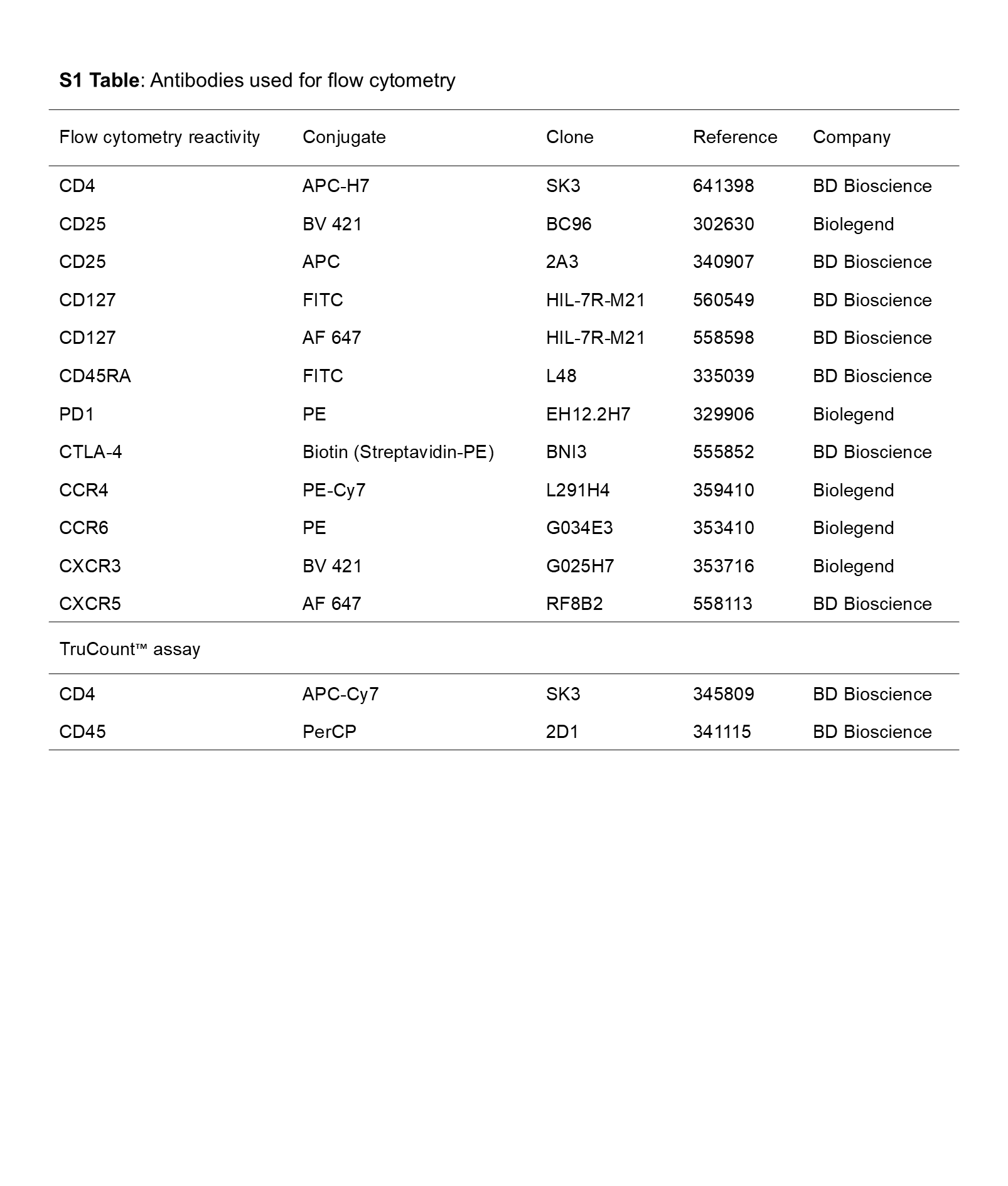

Supplement: S1 Table — (TIF) [file pone.0330823.s001.tif]

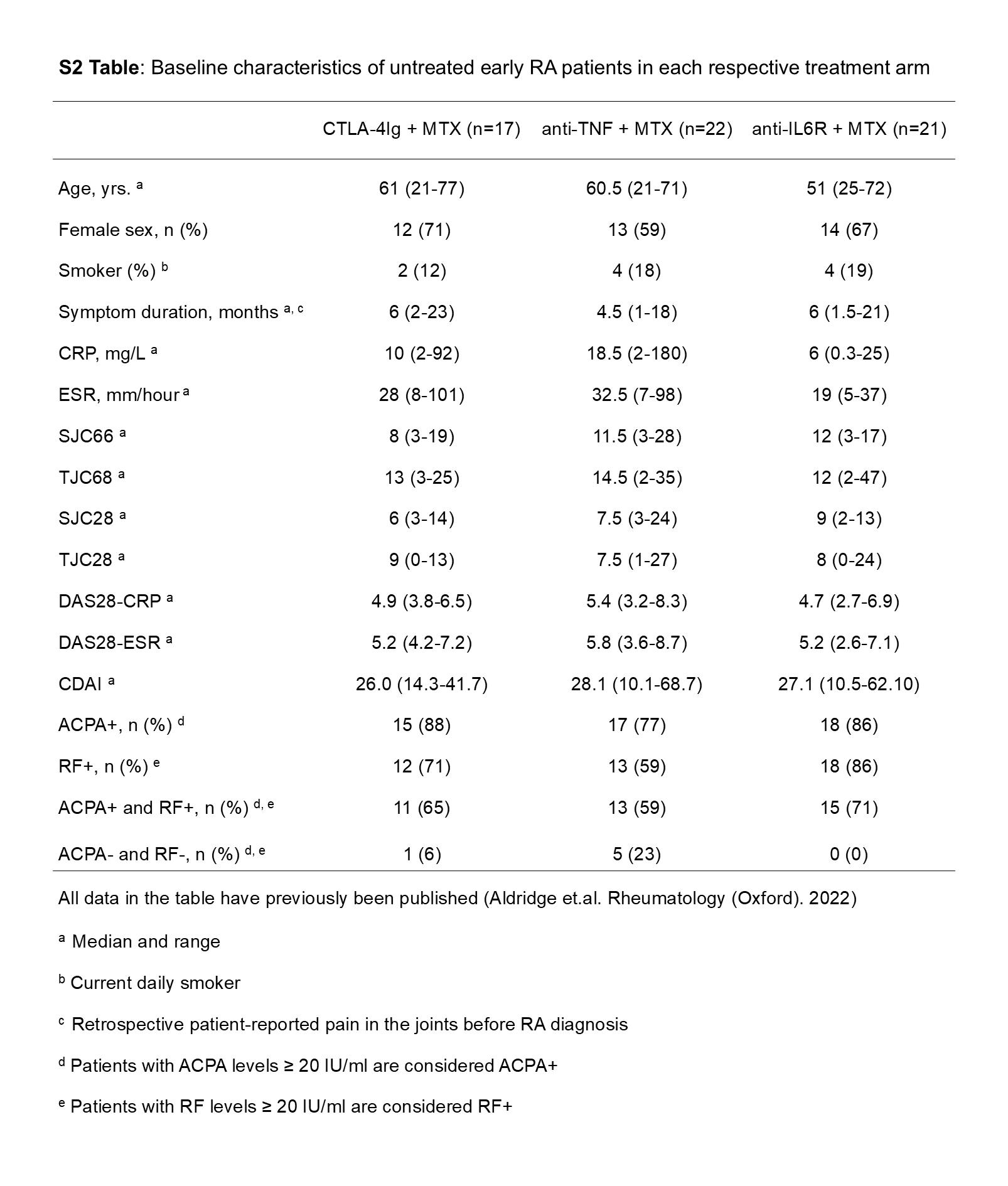

Supplement: S2 Table — (TIF) [file pone.0330823.s002.tif]

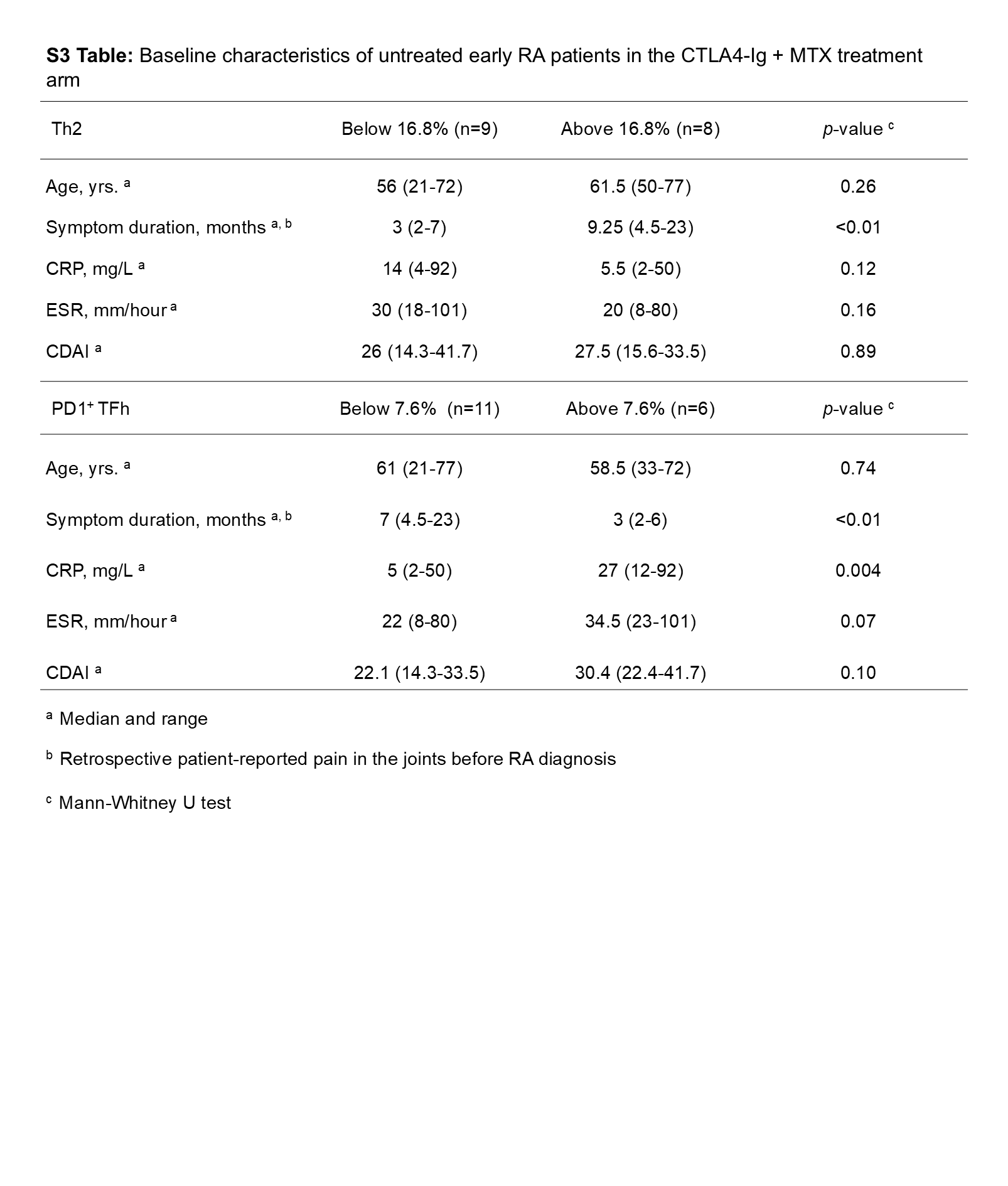

Supplement: S3 Table — (TIF) [file pone.0330823.s003.tif]

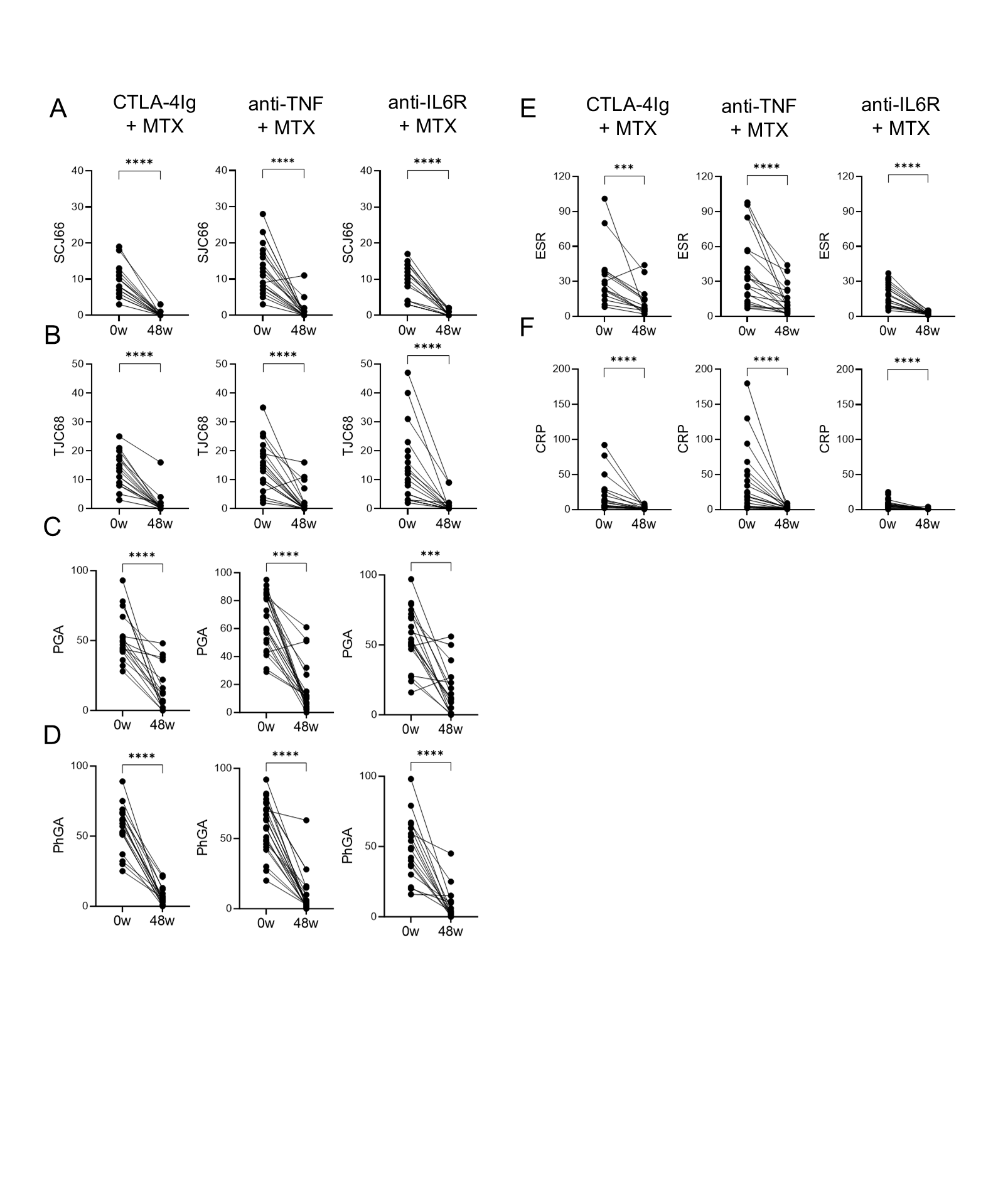

Supplement: S1 Fig — RA disease activity measured by (A) the swollen joint count 66 (SJC66), (B) tender joint count 68 (TJC68), (C) patient global assessment (PGA), (D) physician global assessment (PhGA), (E) ESR, and (F) CRP at baseline (0w) and at 48 weeks (48w) in patients treated with methotrexate (MTX) + CTLA-4Ig (abatacept, n = 17), MTX + anti-TNF (certolizumab-pegol, n = 22), or MTX + anti-IL6 receptor (tocilizumab, n = 21), respectively. Wilcoxon matched-pairs signed rank test ***p ≤ 0.001 and ****p ≤ 0.0001. Patients with missing data for w48 are not included in the pairwise comparisons. (TIF) [file pone.0330823.s004.tif]

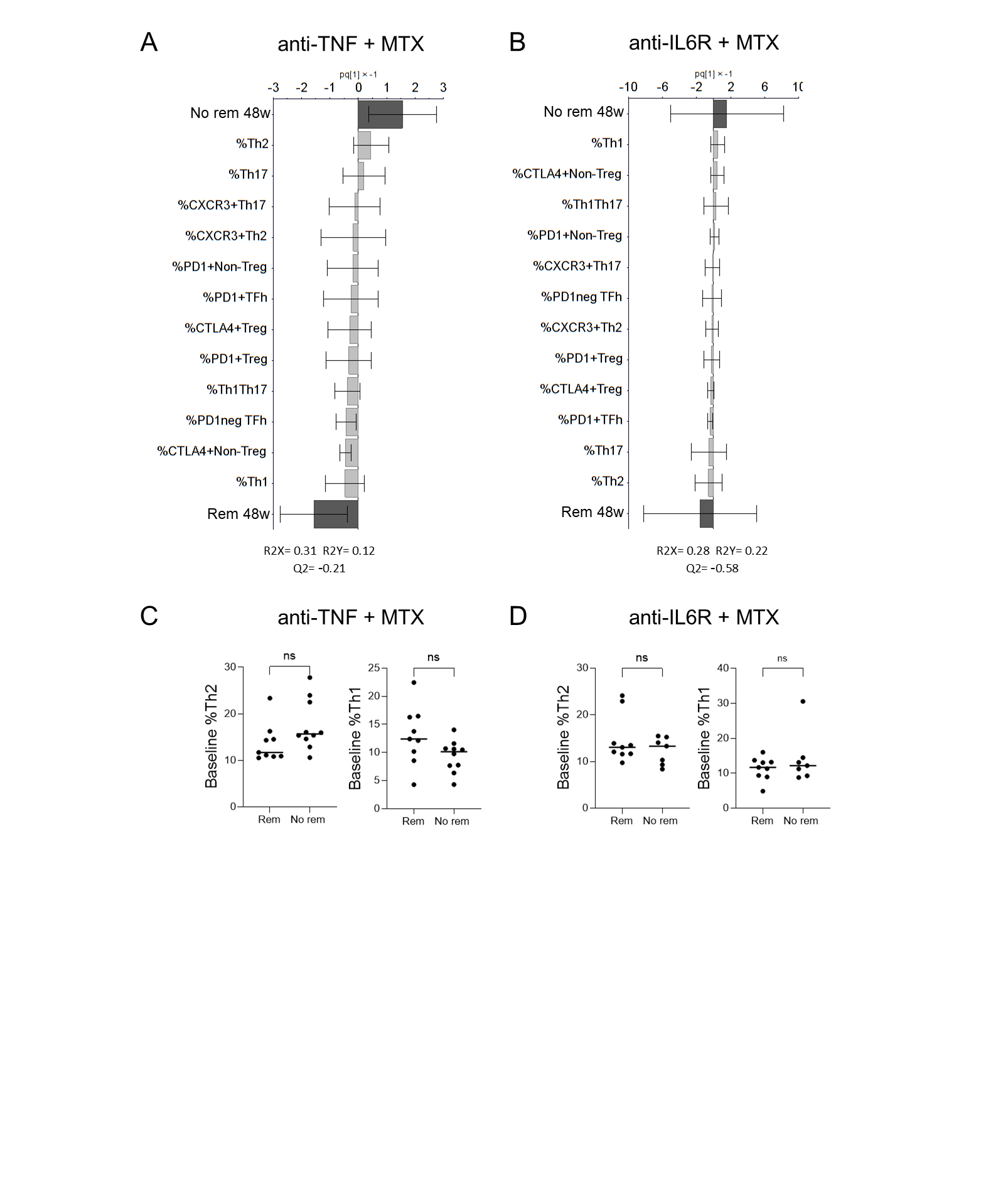

Supplement: S2 Fig — OPLS-DA column loading plots showing the association between remission at week 48 (binary Y-variable) and T-cell subset proportions in blood at baseline (X-variables) in patients treated with (A) methotrexate (MTX) + anti-TNF or (B) MTX + anti-IL6R. (C-D) Comparison of circulating proportions of Th2 and Th1 at baseline in patients who did or did not achieve remission (CDAI ≤ 2.8) at week 48 in the MTX + anti-TNF or MTX + anti-IL6R treatment arm, respectively. Mann–Whitney U-test. Bars indicate median. Two patients with missing data in the MTX + anti-TNF group and six patients with missing data in the MTX + anti-IL6R group were excluded from the analyses. (TIF) [file pone.0330823.s005.tif]

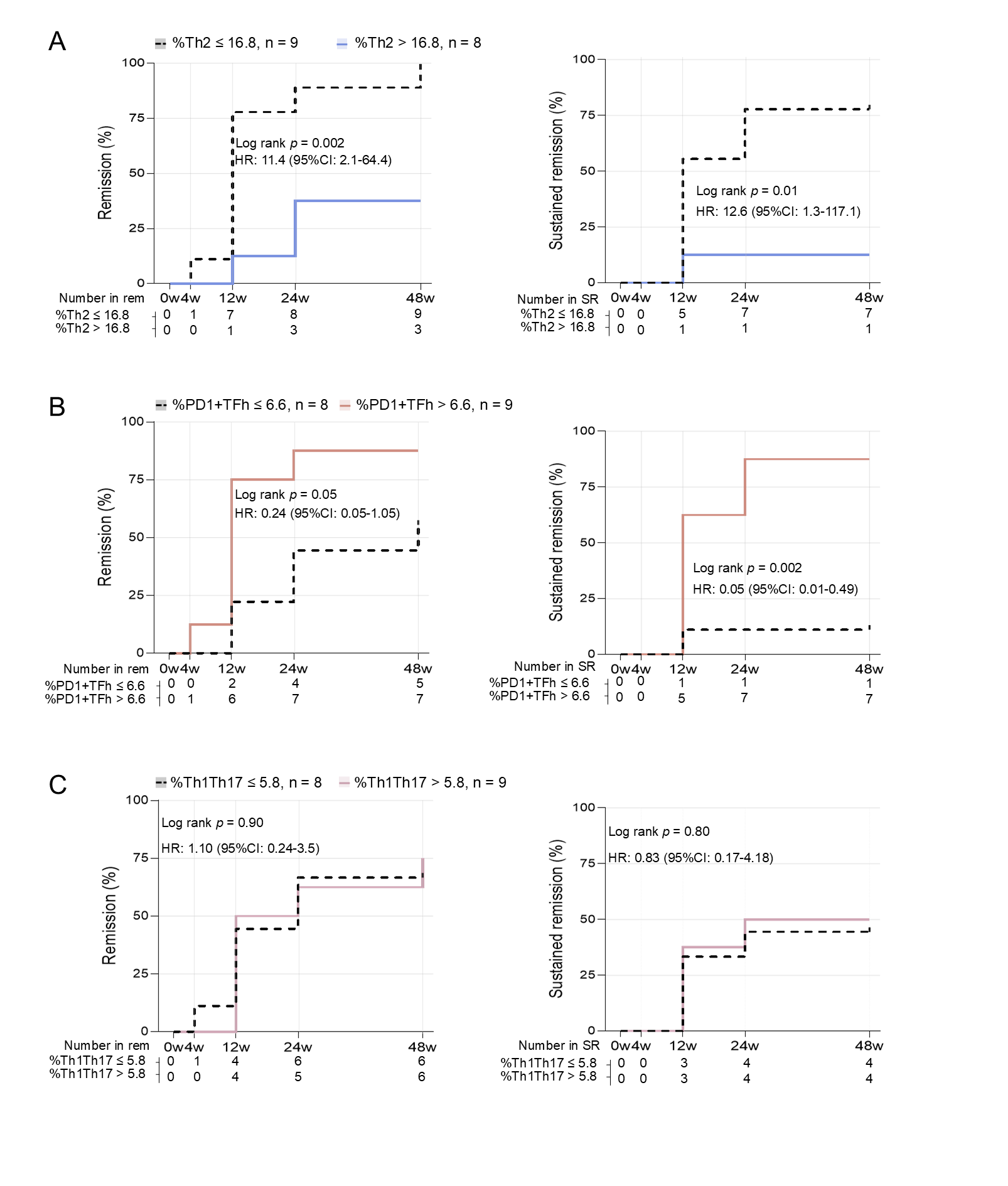

Supplement: S3 Fig — Baseline CD4 + T cell subset proportions are associated with time to achieve remission in CTLA-4Ig-treated patients. Kaplan-Meier cumulative incidence analysis showing the percentage of patients in remission and in sustained remission, i.e., CDAI remission for two or more consecutive visits, with (A) Th2 proportions below the median (≤ 16.8%, dotted line) or above (> 16.8%, blue line) or (B) PD1+ TFh proportions below median (≤ 6.6%, dotted line) or above (> 6.6%, orange line), and (C) Th1Th17 proportions below median (≤ 5.8%, dotted line) or above (> 5.8%, pink line) or at baseline. Significance was tested with the log-rank test, and unadjusted hazard ratios (HR) for achieving remission were estimated using Cox regression. Reference groups were patients with Th2, PD1+ TFh, or Th1Th17 proportions below the median. 95%CI: 95 percent confidence interval. Each Cox regression model fulfilled the proportional hazards assumption. (TIF) [file pone.0330823.s006.tif]

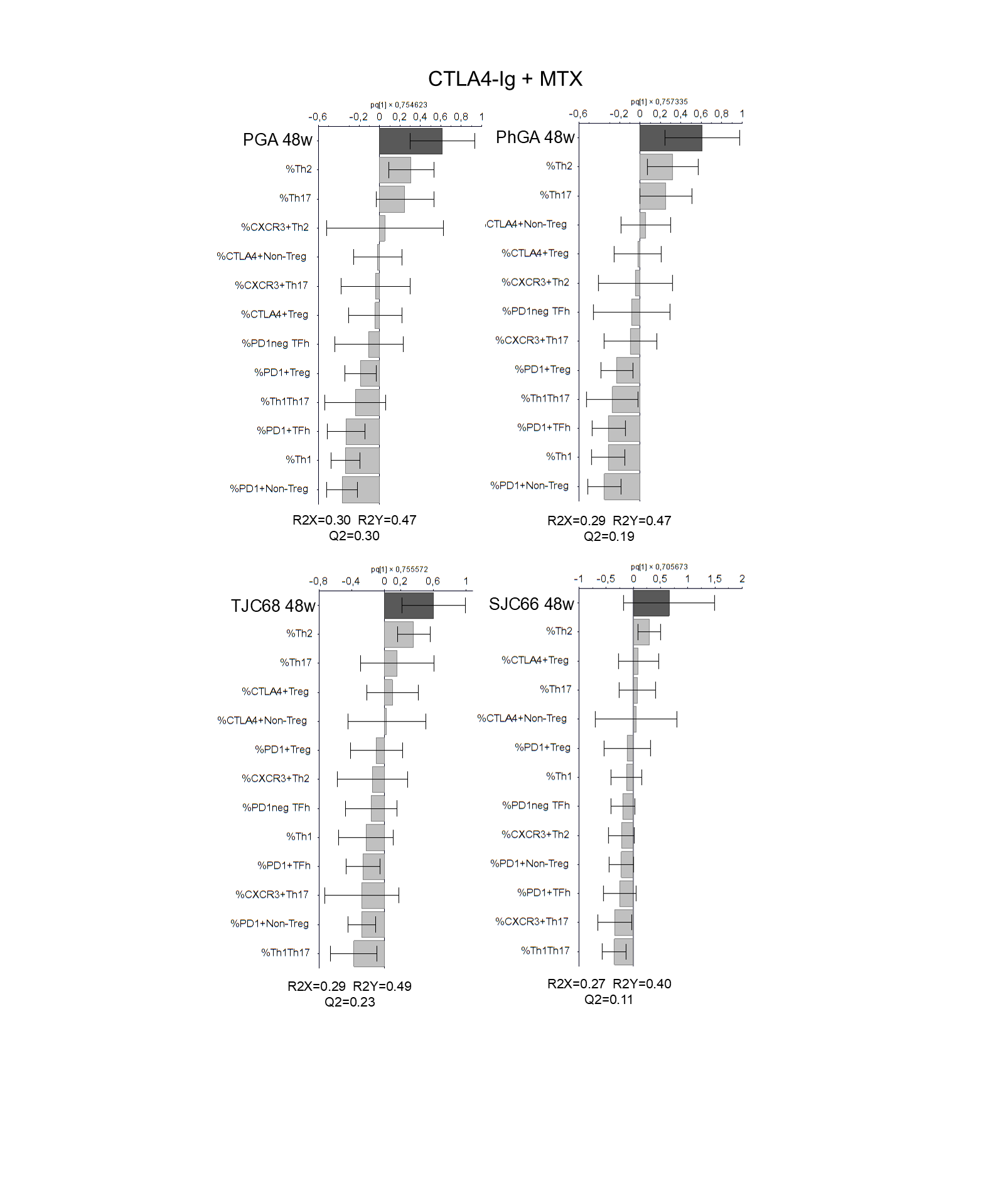

Supplement: S4 Fig — OPLS-DA column loading plots showing the association between patient global assessment (PGA), physician global assessment (PhGA), tender joint count-68 (TJC68) or swollen joint count-66 (SJC66) at 48w (Y-variables) and T-cell subset proportions in blood at baseline (X-variables) in patients treated with methotrexate (MTX) + CTLA-4Ig. (TIF) [file pone.0330823.s007.tif]

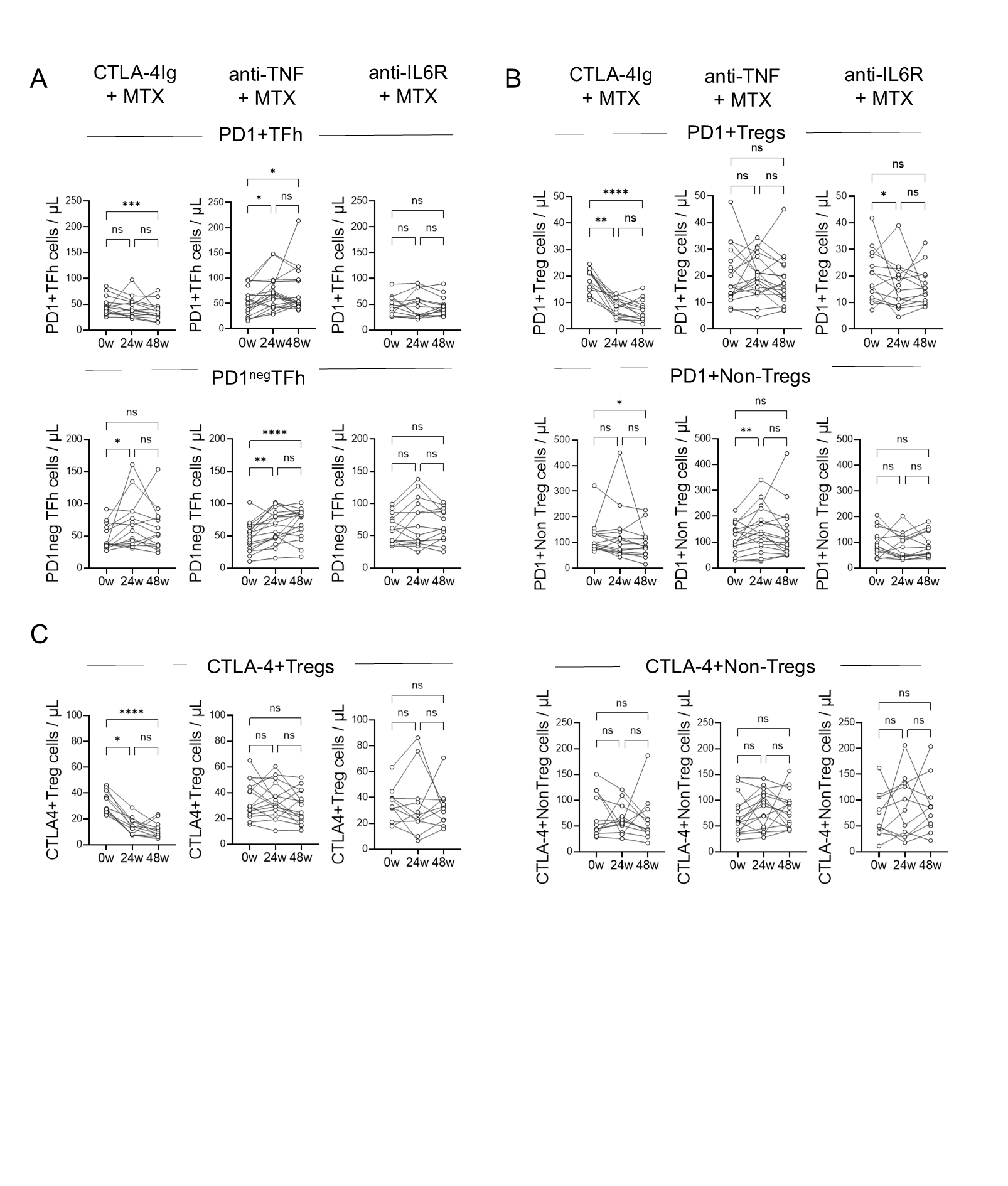

Supplement: S5 Fig — Comparison of the cell count per µL blood of (A) PD1+ TFh and PD1neg TFh, (B) PD1-expressing conventional CD4+ T cells (Non-Tregs, excluding TFh) and regulatory T cells (Tregs), and (C) CTLA-4-expressing Tregs and Non-Tregs at baseline (0w), at 24 weeks, and 48 weeks in patients treated with methotrexate (MTX) + CTLA-4Ig (abatacept, n = 17), MTX + anti-TNF (certolizumab-pegol, n = 22), or MTX + anti-IL6 receptor (tocilizumab, n = 21), respectively. Friedman’s test with Dunn’s test for multiple comparisons. *p ≤ 0.05, **p ≤ 0.01, ***p ≤ 0.001 and ****p ≤ 0.0001. (TIF) [file pone.0330823.s008.tif]

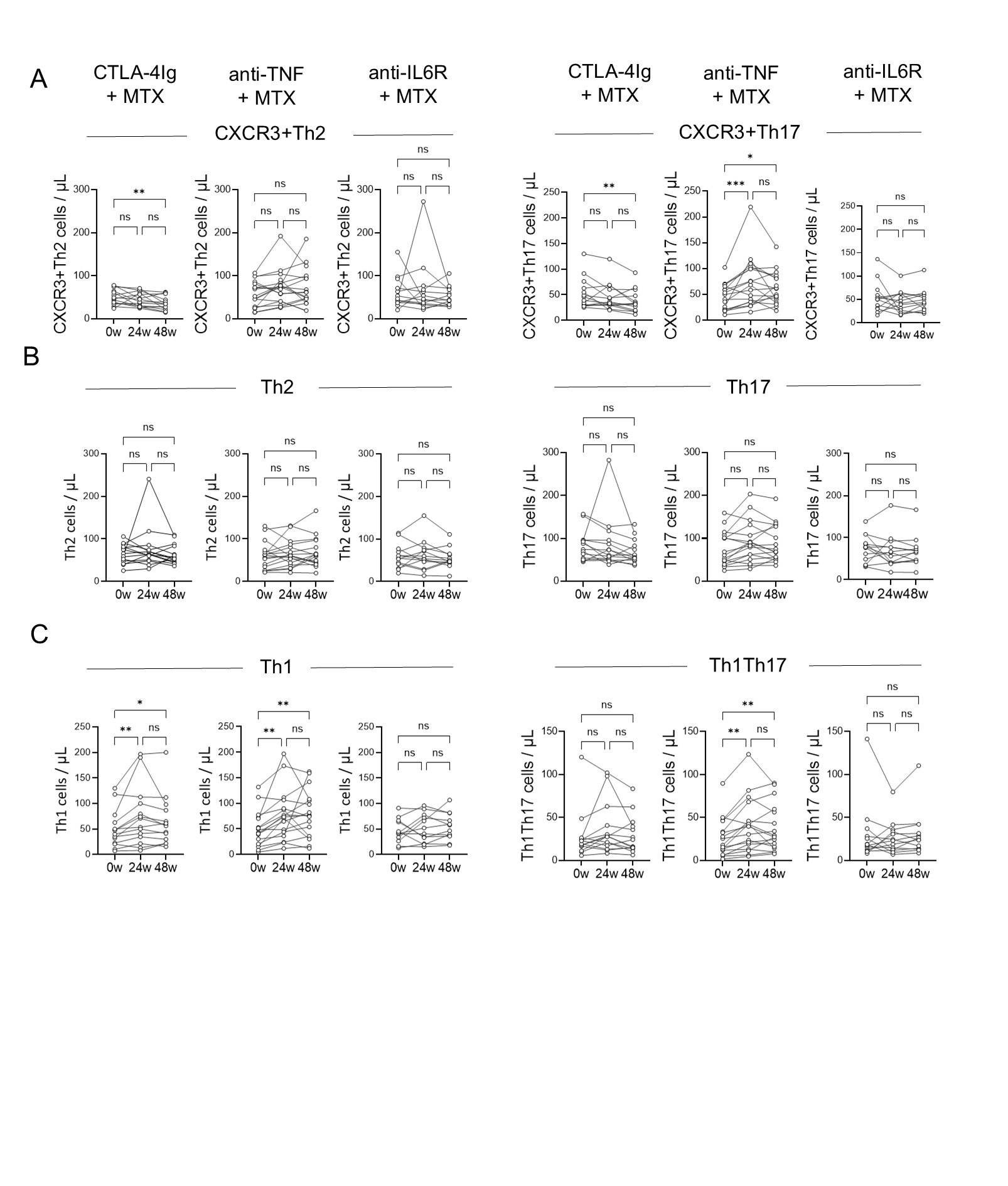

Supplement: S6 Fig — Comparison of the cell count per µL blood of (A) CXCR3+ Th2 and CXCR3+ Th17, (B) Th2 and Th17, and (C) Th1 and Th1Th17 at baseline (0w), at 24 weeks and 48 weeks in patients treated with methotrexate (MTX) + CTLA-4Ig (abatacept, n = 17), MTX + anti-TNF (certolizumab-pegol, n = 22), or MTX + anti-IL6 receptor (tocilizumab, n = 21), respectively. Friedman’s test with Dunn’s test for multiple comparisons. *p ≤ 0.05, **p ≤ 0.01 and ***p ≤ 0.001. (TIF) [file pone.0330823.s009.tif]

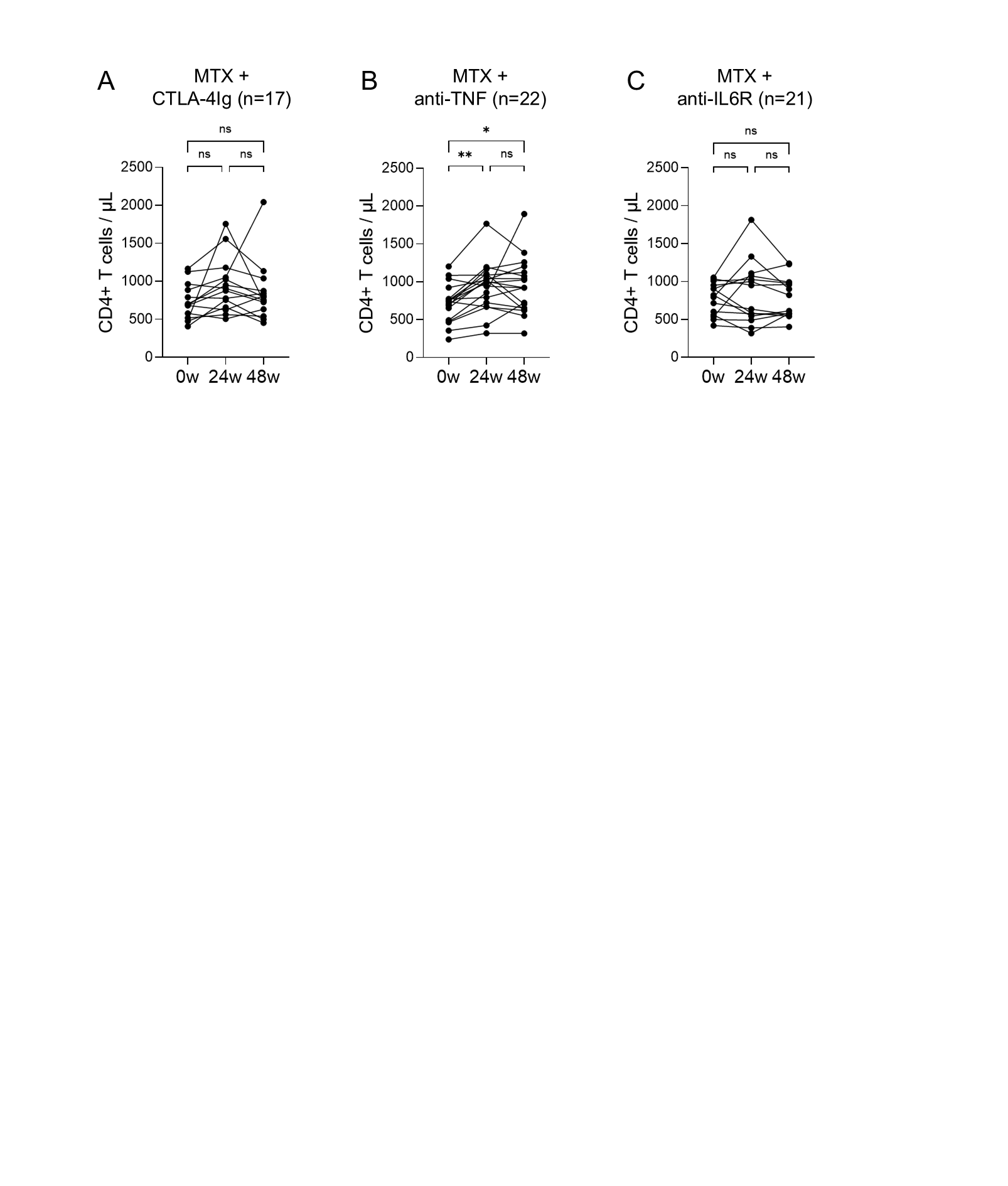

Supplement: S7 Fig — Comparison of the absolute counts of CD4+ T cells at baseline and at 24 and 48 weeks in the blood of patients treated with (A) methotrexate (MTX) + CTLA-4Ig (abatacept, n = 17), (B) MTX + anti-TNF (certolizumab-pegol, n = 22), or (C) MTX + anti-IL6 receptor (tocilizumab, n = 21). Friedman’s test with Dunn’s test for multiple comparisons. *p ≤ 0.05 and **p ≤ 0.01. (TIF) [file pone.0330823.s010.tif]
